# Establishment of an oral enterovirus 71 (EV71) infection model in immunocompetent mice for antiviral therapy evaluation

**DOI:** 10.1128/jvi.02068-25

**Published:** 2026-05-21

**Authors:** Yisha Ma, Dan Luo, Xianliang Ke, Xiaohui Song, Mengchan Hao, Jianjun Chen, Yuan Zhang

**Affiliations:** 1 State Key Laboratory of Virology and Biosafety, Chinese Academy of Sciences Wuhan Institute of Virology 74614 , Wuhan, People's Republic of China; 2 University of Chinese Academy of Sciences 74519, Beijing, People's Republic of China; 3 Department of Gastroenterology, Wuhan Children’s Hospital, Tongji Medical College, Huazhong University of Science and Technology 12403, Wuhan, People's Republic of China; 4 Department of Obstetrics, Wuhan Children’s Hospital, Wuhan Maternal and Child Healthcare Hospital, Tongji Medical College, Huazhong University of Science & Technology 12443, Wuhan, China; University of North Carolina at Chapel Hill, Chapel Hill, North Carolina, USA

**Keywords:** EV71, oral infection, immunocompetent mice, virus transmission *in vivo*, immune response, antiviral drugs, intestinal probiotic blockade therapy

## Abstract

Enterovirus 71 (EV71) is one of the major pathogens responsible for hand-foot-and-mouth disease in children and is associated with severe neurological complications, including death. Despite the fact that EV71 naturally infects humans through the oral route, the absence of a simple and reliable oral infection model has significantly impeded investigations into its pathogenesis and the development of antiviral interventions. In this study, we established a robust oral EV71 infection model in widely used immunocompetent BALB/c mice, employing a clinical isolate devoid of adaptive mutations. Mice infected via the oral route developed disease progression and clinical, as well as pathological features that closely recapitulate those observed in human infection. We further identified marked differences in viral dissemination and host immune responses between oral and intraperitoneal inoculation, underscoring the importance of physiologically relevant oral infection models for elucidating EV71 pathogenesis. In addition, the anti-EV71 candidate drug rupintrivir showed reduced efficacy in the oral infection model compared with the intraperitoneal model. Notably, this oral model enables evaluation of therapies designed to block intestinal viral infection—a capability lacking in intraperitoneal models. Using this platform, we assessed an intestinal probiotic blockade therapy for the first time. Our findings demonstrate that the probiotic formulation effectively suppresses viral replication and alleviates pathological damage in the spleen, lungs, kidneys, and other organs, providing a scientific basis for developing probiotic-based therapeutic strategies against viral infections.

**IMPORTANCE:**

The restricted host tropism of enterovirus 71 (EV71) has limited the development of animal models that accurately recapitulate human disease. Here, we demonstrate for the first time that the clinically isolated EV71-GZCII strain successfully infects 14-day-old immunocompetent BALB/c mice via oral gavage, establishing a physiologically relevant model that addresses this critical gap. Orally infected mice displayed distinct viral dissemination patterns and host immune responses compared with intraperitoneal models, highlighting the critical role of infection route in EV71 pathogenesis. Using this system, we evaluated the *in vivo* therapeutic efficacy of the 3C protease inhibitor rupintrivir and found that its apparent efficacy is strongly influenced by the route of infection. Furthermore, we employed this oral model to assess probiotic-based antiviral therapy, revealing effective suppression of viral replication and pathology. Unlike intraperitoneal systems, this model uniquely enables evaluation of intestinal interventions, providing a robust platform for EV71 research and therapy development.

## INTRODUCTION

Enterovirus 71 (EV71) is a common cause of hand-foot-and-mouth disease (HFMD) in infants and children under 5 years of age. While most cases present mild symptoms, EV71 infection can lead to severe neurological complications and myocarditis, which account for the majority of HFMD-related deaths (1–3). Epidemiologically, EV71 emerged as a global health threat following its widespread outbreak across the Asia-Pacific region, with notable epidemics in Malaysia (1997), Singapore (2000), and China (2008) (4–10). Current surveillance data indicate that EV71 is a predominant neurotropic enterovirus associated with the cyclical resurgence of HFMD outbreaks worldwide. Recurrent epidemics with associated fatalities have been reported in multiple countries, including Thailand (11), Japan (12), Malaysia (13), Vietnam (14), China (15), and Indonesia (16), occurring annually or every 2–3 years. Although EV71 primarily affects infants and children under 5 years of age, an increasing number of adult cases have been reported in China in recent years (17–21). This apparent rise may partially reflect heightened public health awareness and improvements in surveillance and diagnostic capacity, resulting in increased detection and reporting. In addition, the emergence of viral genetic variants associated with enhanced pathogenic potential may also contribute to this trend. However, the lack of robust animal infection models hindered comprehensive elucidation of EV71 pathogenesis, and no antiviral agents have yet been approved for its treatment.

The development of effective animal models for EV71 has faced persistent challenges due to the virus’s strict human tropism and unique pathogenesis. Under natural conditions, EV71 primarily spreads via the fecal-oral route, infecting humans through intestinal invasion (9, 22–24). However, due to the absence of an effective oral infection model, most mouse models used to study EV71 pathogenesis rely on intraperitoneal injection (25–33). This approach evades the natural infection process and neglects the critical role of the intestinal immune barrier, thereby precluding investigation of viral transmission and infection mechanisms within the gut, hindering the development of intestinal blockade therapies, and limiting the reliability and applicability of experimental findings. Therefore, establishing an oral EV71 infection mouse model is crucial for elucidating pathogenesis and developing effective therapeutic strategies.

To date, the establishment of physiologically relevant oral infection models for EV71 remains limited. Existing models primarily rely on mouse-adapted strains, transgenic mice, or immunocompromised mice (22, 28, 34). For instance, Ya-Fang Wang et al. (22) and Elizabeth A. Caine et al. (35) developed a 1-day-old ICR mouse model and a 7-day-old AG129 mouse model using a mouse-adapted strain of EV71. Nonetheless, these mouse-adapted strains accumulate multiple mutations, which do not accurately reflect the infection characteristics of authentic clinical isolates. The knock-in of the hSCARB2 gene enhances EV71 susceptibility in mice, enabling the establishment of oral infection models in hSCARB2 transgenic mice (36). Despite this, hSCARB2 transgenic models are associated with high costs and suboptimal infection efficiency, limiting their broader application. Moreover, the expression pattern of hSCARB2 can profoundly influence viral susceptibility and tissue tropism (25). Although the overall expression profile of hSCARB2 in transgenic mice resembles that in humans, tissue-specific expression affinity is not fully identical, and hSCARB2 expression may also vary with the age of the mice (26). Additionally, immunocompromised mice have been employed in EV71 oral infection models, such as the F23 strain in 3-day-old NOD/SCID mice (34) and the strain 41 in 2-week-old AG129 mice (29). Nevertheless, these models fail to fully capture the complex interactions between host immunity and viral infection. Although these animal models have advanced our understanding of EV71 infection and pathogenesis, their limitations must be considered when interpreting results. Therefore, it is important to develop an effective oral infection model using immunocompetent mice inoculated with clinical EV71 strains.

The EV71-GZCII strain, isolated from hospitalized children with HFMD, exhibited high pathogenic properties in mice (3). Using this clinical strain, we successfully established an oral infection model in widely used immunocompetent BALB/c mice, rather than in immunodeficient or transgenic mice as previously reported. Comparative analyses revealed marked differences in viral dissemination dynamics and host antiviral immune responses between the oral (intragastric, i.g.) and intraperitoneal (i.p.) infection models, underscoring the importance of physiologically relevant oral models for EV71 research. Using this model, we evaluated the efficacy of rupintrivir, one of the anti-EV71 candidates. Moreover, unlike intraperitoneal models that sidestep the gastrointestinal tract, the oral model captures the critical role of the intestinal barrier in shaping antiviral responses. Leveraging this advantage, we further assessed an intestinal probiotic blockade therapy against EV71 infection. Gavage delivery of probiotics demonstrated notable antiviral activity, effectively suppressing viral replication and associated pathology. Collectively, these findings establish a robust platform and provide translational insights for the clinical management of EV71.

## RESULTS

### Oral inoculation with EV71-GZCII exhibited potent pathogenicity and high mortality in immunodeficient mice

**Fig 1:**
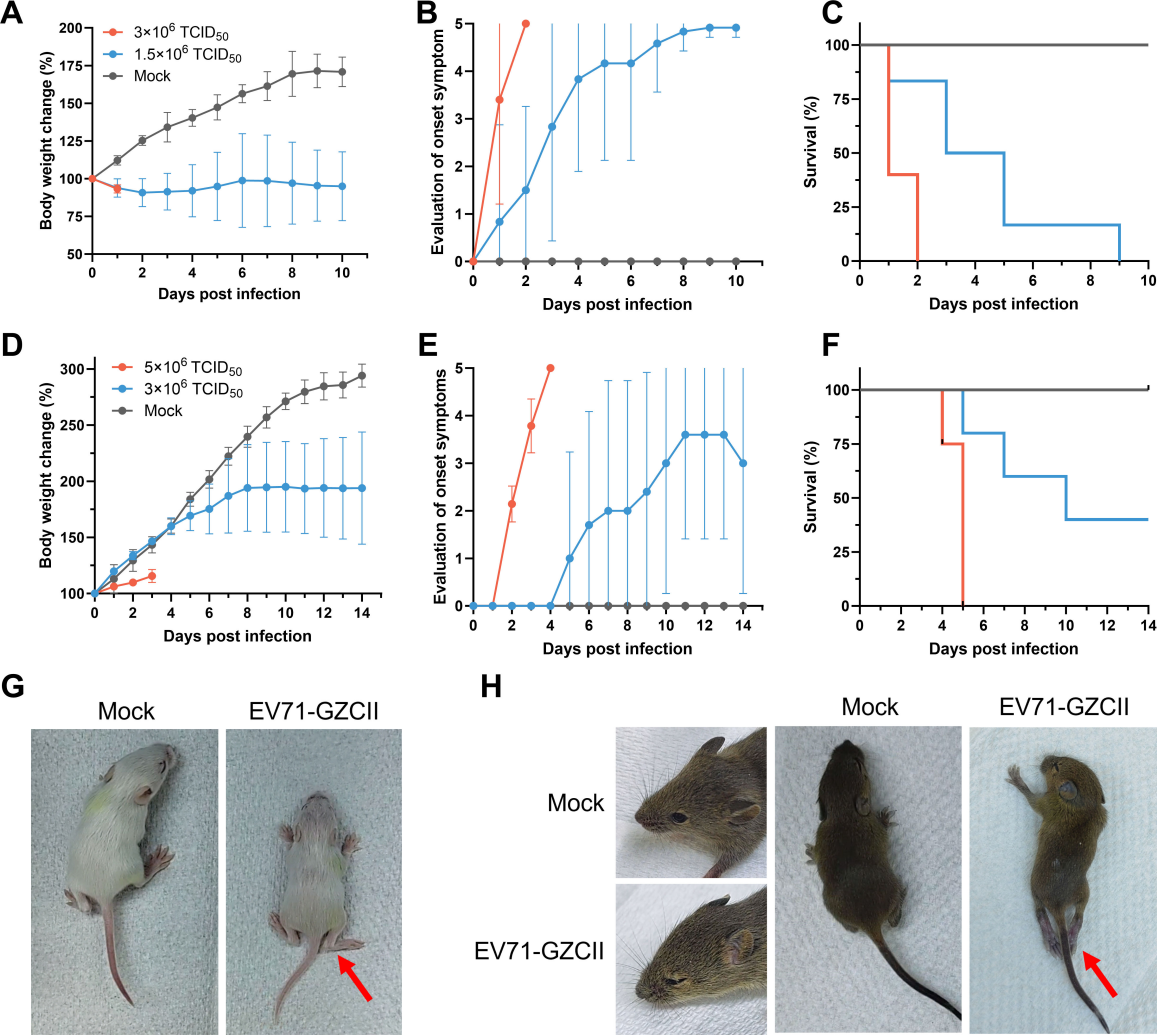
Oral infection of EV71-GZCII in immunodeficient murine models. Body weight change rate (**A**), clinical symptom score (**B**), and survival curve (**C**) in 7-day-old NOD/SCID mice orally infected with EV71-GZCII. Virus doses were 1.5 × 10⁶ TCID₅₀ (*n* = 6) and 3 × 10⁶ TCID₅₀ (*n* = 5). Mock-infected controls were administered PBS (*n* = 6). Body weight change rate (**D**), clinical symptom score (**E**), and survival curve (**F**) in 7-day-old A129 mice orally infected with EV71-GZCII. Virus doses were 3 × 10⁶ TCID₅₀ (*n* = 5) and 5 × 10⁶ TCID₅₀ (*n* = 4). Mock-infected controls received PBS (*n* = 5). (**G**) Representative clinical manifestation of early-stage neuropathogenesis in EV71-infected NOD/SCID mice, demonstrating acute limb paralysis (arrow). (**H**) Pathological features in A129 mice, including ocular irritation and progressive hindlimb paralysis (arrow).

To better reappear the natural infection process of EV71, we sought to establish an oral infection mouse model using the highly pathogenic clinical isolate EV71-GZCII. Although EV71 infection models have been successfully developed in NOD/SCID and AG129 mice (32, 34, 35), their oral infection efficiency remains relatively low. Therefore, we first evaluated these immunodeficient mice to determine the oral infectivity of EV71-GZCII. For the oral infection model, 7-day-old NOD/SCID mice were inoculated with 1.5 × 10⁶ TCID₅₀ of EV71-GZCII. This dose induced progressive mortality, with weight loss detectable as early as 1 day post-infection (dpi) (Fig. 1A), accompanied by progressively worsening clinical symptoms (Fig. 1B). Mortality was observed at 1, 3, and 5 dpi, with all mice succumbing by 9 dpi. A higher dose of 3 × 10⁶ TCID₅₀ resulted in rapid systemic collapse, characterized by sudden weight loss and acute exacerbation of clinical symptoms (Fig. 1A and B), achieving 100% mortality within 2 days (Fig. 1C). The acute lethality was associated with early-onset neurological deficits, including paralysis and motor dysfunction (Fig. 1G).

In parallel experiments, A129 mice displayed strain-specific susceptibility patterns. Mice orally infected with 3 × 10⁶ TCID₅₀ of EV71-GZCII failed to gain weight by 8 dpi (Fig. 1D), exhibited clinical symptoms starting at 4 dpi (Fig. 1E), and experienced a 60% mortality rate (Fig. 1F). A higher dose of 5 × 10⁶ TCID₅₀ caused acute exacerbation of clinical symptoms (Fig. 1E) and complete lethality by 5 dpi (Fig. 1F). Notably, significant ocular irritation and limb paralysis were observed in the A129 mice (Fig. 1H). These results indicate that EV71-GZCII exhibited significantly higher infection efficiency, achieving 100% mortality in both NOD/SCID and AG129 mice under oral infection conditions.

### The EV71-GZCII strain exhibited potent oral pathogenicity in immunocompetent BALB/c mice through dose- and age-dependent characteristics

**Fig 2:**
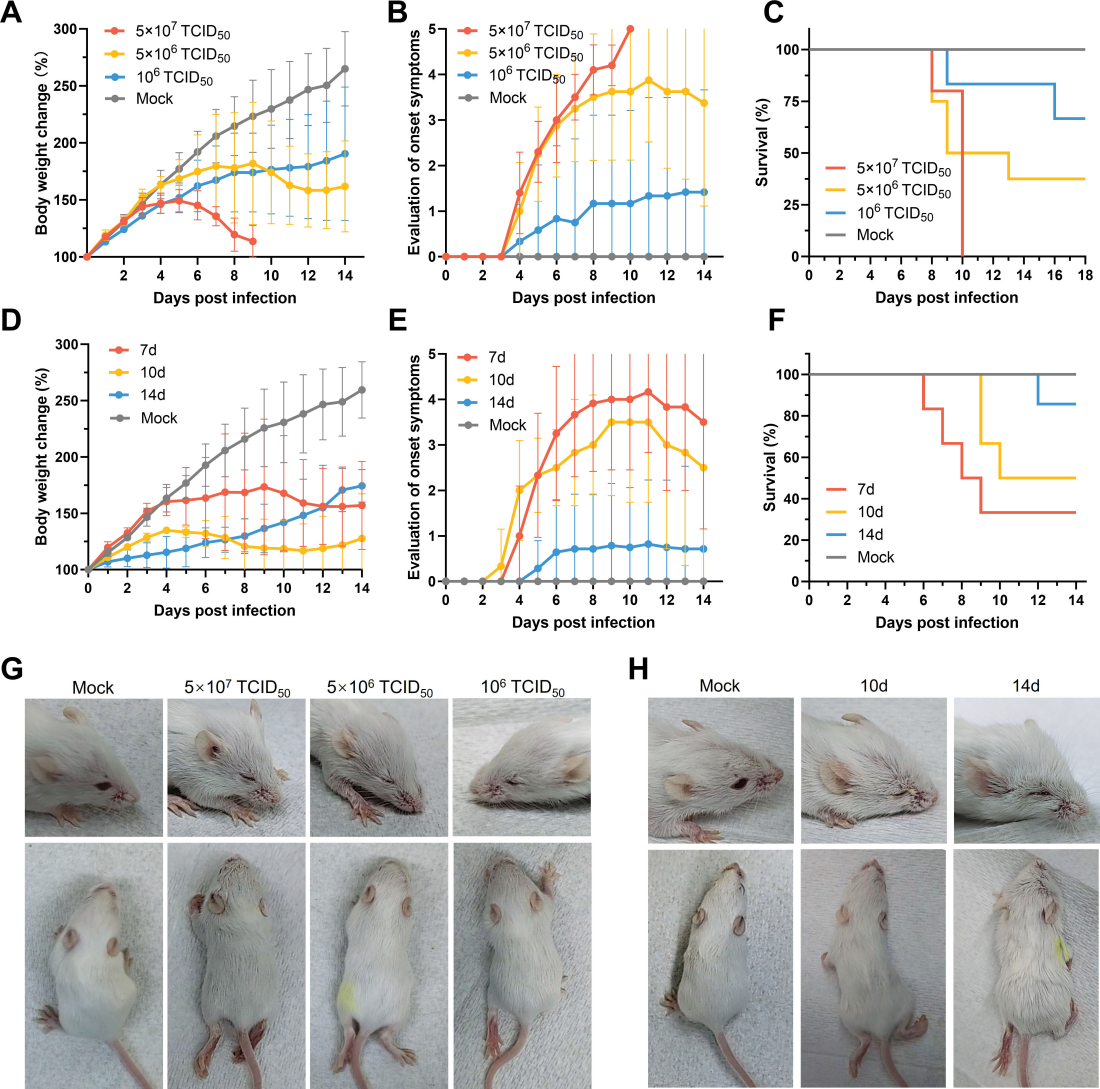
Dose-response relationship and age-dependent susceptibility to EV71-GZCII infection in immunocompetent BALB/c mice. Body weight change rate (**A**), clinical symptom score (**B**), and survival curve (**C**) in 7-day-old BALB/c mice orally infected with EV71-GZCII. Virus doses were 1 × 10⁶ TCID₅₀ (*n* = 6), 5 × 10⁶ TCID₅₀ (*n* = 8), or 5 × 10⁷ TCID₅₀ (*n* = 5). Mock-infected controls were administered PBS (*n* = 6). Body weight change rate (**D**), clinical symptom score (**E**), and survival curve (**F**) in 7-day-old (*n* = 6), 10-day-old (*n* = 6), and 14-day-old (*n* = 14) BALB/c mice orally infected with 5 × 10⁶ TCID₅₀ EV71-GZCII. (**G**) Pathognomonic signs in 7-day-old BALB/c mice, including ocular irritation and progressive hindlimb paralysis. (**H**) Neurological manifestations in EV71-infected mice, including ocular irritation and progressive hindlimb paralysis.

After confirming the potent pathogenicity of EV71-GZCII via oral infection, we challenged 7-day-old BALB/c mice with three distinct viral titers: 10⁶, 5 × 10⁶, and 5 × 10⁷ TCID₅₀ via oral gavage. Mock-treated controls received 50 μL PBS. Infected mice exhibited progressive weight loss starting from 5 dpi (Fig. 2A). Initial symptoms, including piloerection and reduced activity, appeared at 4 dpi and progressed to limb paralysis and death (Fig. 2B). The dose of 5 × 10⁷ TCID₅₀ achieved 100% mortality by 10 dpi, with the first fatalities occurring at 8 dpi. The doses of 5 × 10⁶ and 10⁶ demonstrated 62.5% and 33.33% mortality rates, respectively (Fig. 2C). All infected groups developed neuropathological features, including ascending flaccid paralysis and bilateral ocular inflammation (Fig. 2G), recapitulating key complications observed in human HFMD.

To assess age-related susceptibility, 7-, 10-, and 14-day-old BALB/c mice were orally challenged with 5 × 10⁶ TCID₅₀ of EV71-GZCII. Weight loss in 7-day-old mice began at 9 dpi, while 10-day-old mice exhibited earlier weight decline (starting at 4 dpi), reaching a nadir (116.77% of initial weight) at 11 dpi, followed by recovery. Two out of 14 of the 14-day-old mice displayed sub-baseline weights (80.09% and 76.84%) with overall growth retardation (Fig. 2D). Clinical scores peaked in 7-day-old mice (4.17 at 4 dpi), followed by 10-day-old mice (3.50 at 3 dpi) and 14-day-old mice (0.82 at 5 dpi; Fig. 2E). Mortality rates decreased with increasing age, with 66.67% (6–9 dpi) in 7-day-old, 50% (9–10 dpi) in 10-day-old, and 14.29% (12 dpi) in 14-day-old mice (Fig. 2F). All infected groups developed ocular irritation and neurological symptoms, such as limb paralysis (Fig. 2H). These results demonstrate an age-dependent susceptibility to EV71-GZCII infection in BALB/c mice, with 14-day-old mice exhibiting markedly lower susceptibility compared to 10-day-old and 7-day-old counterparts.

### EV71-GZCII infection via oral and intraperitoneal routes exhibits markedly distinct organ tropism and systemic dissemination profiles

**Fig 3:**
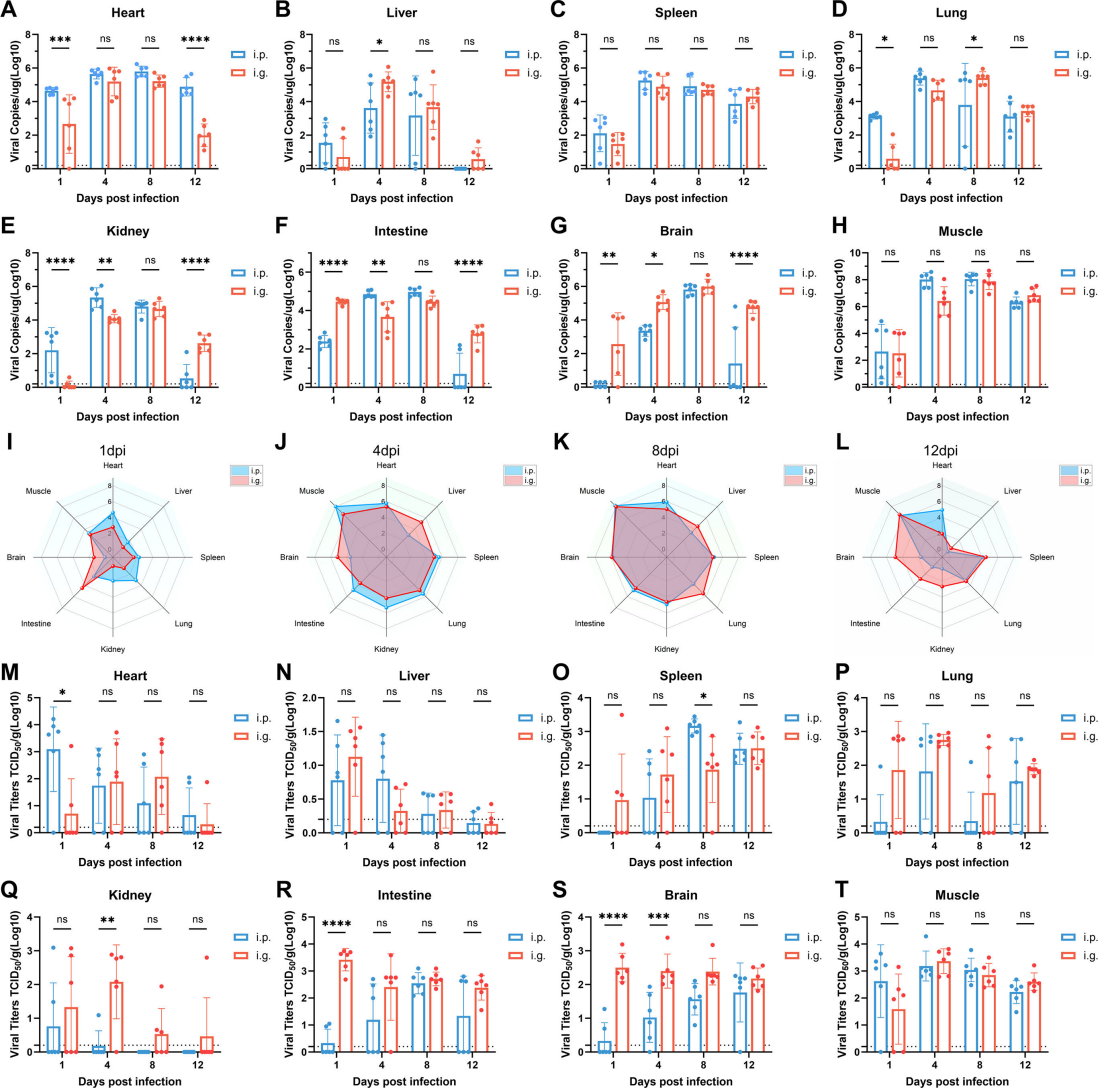
Spatiotemporal dissemination kinetics of EV71-GZCII in BALB/c mice. 7-day-old BALB/c mice were inoculated with 1 LD_50_ of EV71-GZCII via oral (i.g.) or i.p. routes. Tissues were harvested at 1, 4, 8, and 12 dpi for viral RNA quantification by RT-qPCR. (**A–H**) Tissue-specific viral dissemination: (**A**) heart, (**B**) liver, (**C**) spleen, (**D**) lung, (**E**) kidney, (**F**) intestine, (**G**) brain, and (**H**) skeletal muscle. (**I–L**) Temporal viral load progression at (**I**) 1 dpi, (**J**) 4 dpi, (**K**) 8 dpi, and (**L**) 12 dpi. Vertical axis units: viral copies/μg (Log_10_). (**M–T**) Virus titers in (**M**) heart, (**N**) liver, (**O**) spleen, (**P**) lung, (**Q**) kidney, (**R**) intestine, (**S**) brain, and (**T**) skeletal muscle. The dashed line indicates the limit of detection. ns: not significant. *P < 0.05, **P < 0.01, ***P < 0.001, and ****P < 0.0001.

Current EV71 animal models primarily employ intraperitoneal injection, which circumvents the natural route of infection (37, 38). To determine whether the infection route influences viral pathogenesis, we systematically compared viral entry, replication, and dissemination profiles between oral (intragastric, i.g.) inoculation and intraperitoneal (i.p.) injection in 7-day-old BALB/c mice. First, we infected mice with 10-fold serial dilutions of EV71-GZCII via i.g. or i.p. routes, recorded mortality data (Table S1), and calculated the median lethal dose (LD_50_) for each route using the Reed and Muench method. Mice were infected with 1 LD_50_ of EV71-GZCII via either route. Viral RNA loads and infectious titers in major organs were analyzed at 1, 4, 8, and 12 dpi. Six mice per group were euthanized at each time point for analysis (Fig. 3; Fig. S1).

In the oral infection model, the blood and intestines exhibited the highest viral load at 1 dpi (Fig. S1; Fig. 3F, I and R), followed by the muscles and heart (Fig. 3A, H and I), indicating initial viral spread from the inoculation site to the bloodstream and muscles. By 4 dpi, systemic infection was established, with significant viral RNA detected in all tissues. The virus exhibited the strongest tropism for muscle tissue (Fig. 3H, J and T), followed by the heart and brain (Fig. 3A, G, J and S). At 8 dpi, muscles remained the primary site of replication (Fig. 3H, K and T), with increasing tropism for the brain (Fig. 3G, K and S). Viral amplification was also observed in the heart and intestines (Fig. 3A, F, K, M and R), while viral loads in the kidney tissue decreased (Fig. 3E, K and Q). By 12 dpi, viral clearance was observed in all organs, particularly in the liver and heart (Fig. 3A, B, L, M and N), but muscles and brain retained high viral persistence (Fig. 3G, H, L, S and T). In contrast, i.p. infection circumvented intestinal colonization (Fig. 3F, I and R), with the virus entering circulation and initially targeting the blood (Fig. S1), heart (Fig. 3A, I and M), and muscles (Fig. 3H, I and T). Systemic infection was also established by 4 dpi, with muscles being the dominant site of replication (Fig. 3H, J and T). Brain invasion was delayed and less pronounced compared to i.g. infection (Fig. 3G, J and S). At 8 dpi, the muscles and heart remained the primary sites of viral amplification (Fig. 3A, H, K, M and T), with a noticeable increase in viral loads in the brain (Fig. 3G, K and S). However, viral replication declined in the spleen, kidneys, and lungs (Fig. 3C, D, E and K). By 12 dpi, rapid viral clearance occurred in the liver, kidneys, intestines, and brain (Fig. 3B, E, F, G and L), with residual viral loads only in the muscles and heart (Fig. 3A, H and L). These findings reveal route-dependent pathogenesis of EV71. The prolonged viral persistence in muscles and brain during oral infection aligns with clinical manifestations of EV71-associated myositis and encephalitis, underscoring the model’s physiological relevance.

### Comparative pathological analysis of EV71-GZCII infection via oral and intraperitoneal routes

**Fig 4:**
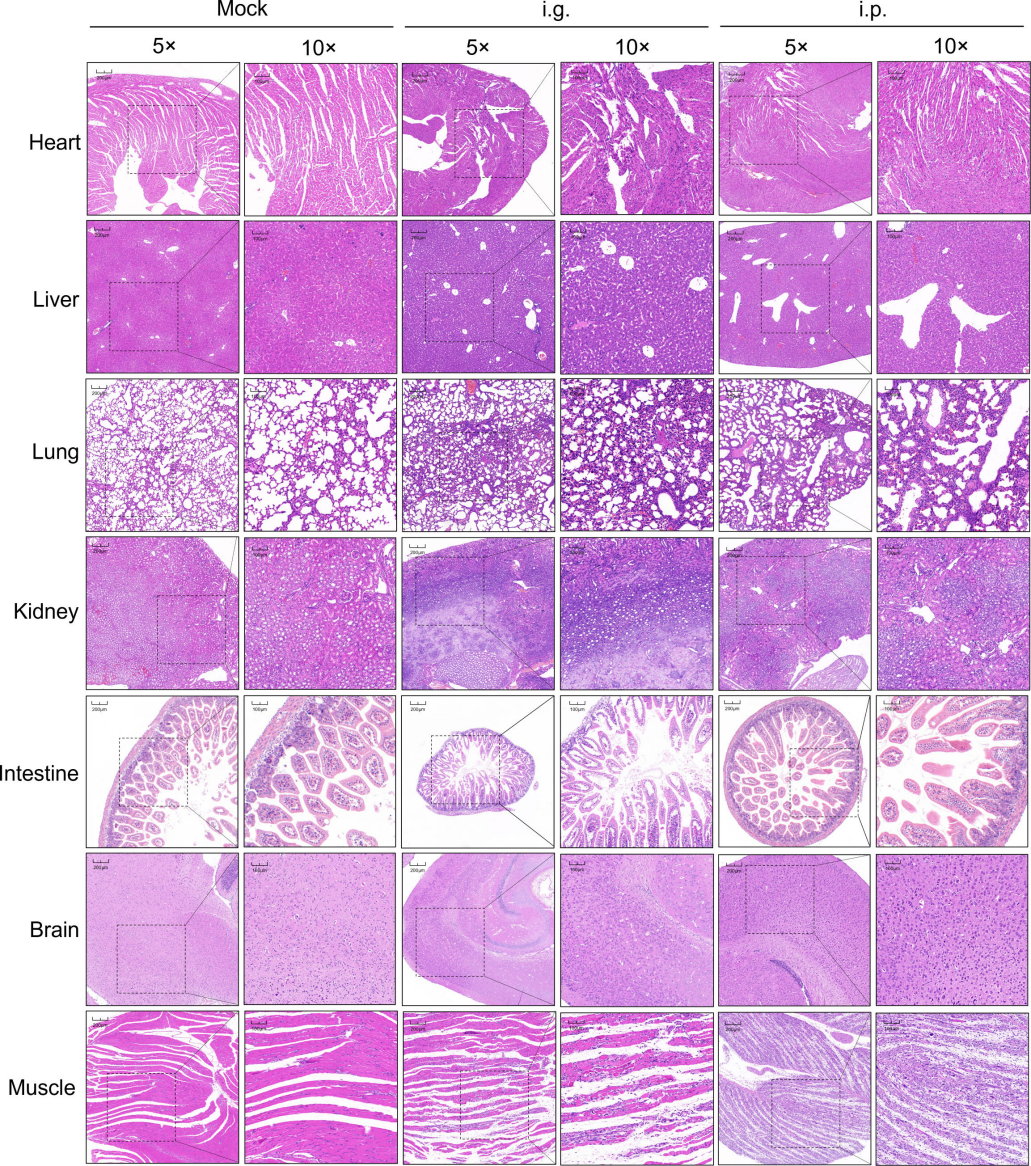
Systemic histopathology induced by EV71-GZCII infection in BALB/c mice. Mock: uninfected mice, i.g.: orally infected mice, i.p.: intraperitoneally infected mice. H&E staining of heart, liver, lung, kidney, intestine, brain, and muscle. Scale bars: 200 μm (5× amplification) and 100 μm (10× amplification).

To evaluate the multi-organ pathology induced by EV71-GZCII, 7-day-old BALB/c mice were inoculated either orally or intraperitoneally with 1 LD_50_ of EV71-GZCII and euthanized at 8 dpi for histopathological assessment. Compared to the uninfected control group (Mock), orally infected mice exhibited significant reductions in spleen length (Fig. S2A) and varying degrees of weight loss in the heart and spleen (Fig. S2B). Splenic atrophy in the infected mice resembles the clinical symptoms observed in children infected with EV71 (39). Tissues, including the heart, liver, lung, kidney, intestine, brain, and muscle, were fixed, embedded in paraffin, sectioned, and stained with hematoxylin and eosin (H&E) for histological examination. Both oral and intraperitoneal EV71-GZCII infections induced multisystemic lesions in mice. In orally infected mice, cardiac tissue displayed tightly arranged myocardial fibers with numerous small round vacuoles, whereas similar abnormalities were also present in intraperitoneally infected mice (Fig. 4). Notably, central vein dilation was more pronounced following intraperitoneal infection, while inflammatory cell infiltration was more severe in orally infected mice (Fig. 4). Pulmonary tissues from both groups exhibited marked pathological changes, including alveolar rupture, prominent thickening of alveolar septa, bronchiectasis, and extensive lymphocytic infiltration (Fig. 4). In the kidneys, cortical and medullary structures were disrupted, with glomerular dissolution accompanied by widespread serous exudation (Fig. 4). Intestinal injury was markedly greater after oral infection than intraperitoneal injection (Fig. 4), likely reflecting direct viral targeting of the gut in the oral route (Fig. 3F). In brain tissues, numerous neurons exhibited degeneration and shrinkage (Fig. 4). Although no significant differences in viral tropism for muscle tissues were detected between infection routes (Fig. 3H), intraperitoneal infection occasionally caused more severe myocyte dissolution. Collectively, these findings indicate that EV71-GZCII can disseminate systemically and invade multiple organs, including the central nervous system (CNS), via oral infection, resulting in widespread tissue damage.

### Intraperitoneal and oral infection of EV71-GZCII elicits different immune responses in the host

**Fig 5:**
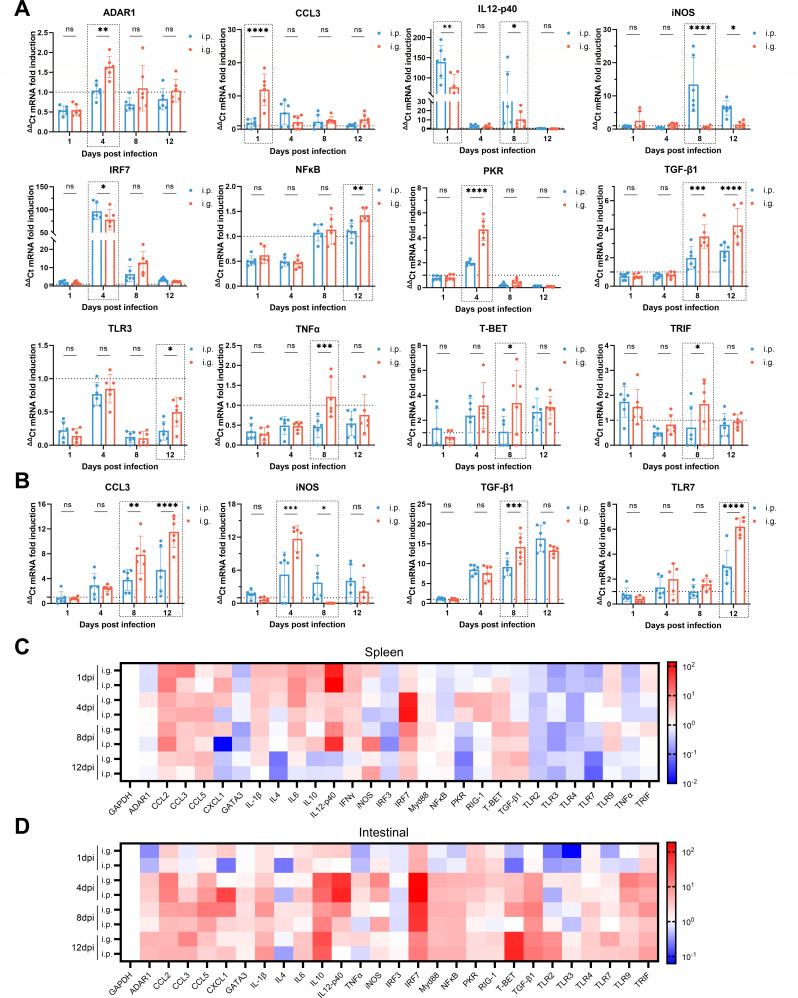
Splenic and intestinal cytokine dysregulation following EV71-GZCII infection. Temporal cytokine mRNA profiles in the spleen (**A**) and intestine (**B**) of BALB/c mice inoculated via i.g. or i.p. routes (1 LD_50_). Relative expression levels of splenic and intestinal cytokines normalized to GAPDH (2^−∆∆Ct^ method). The dashed line indicates baseline levels (mock-uninfected controls). Heatmap visualization of log_10_-transformed fold-changes in splenic (**C**) and intestinal (**D**) tissue across different infection routes and timepoints (1, 4, 8, and 12 dpi). The color gradient reflects transcriptional upregulation (red) or suppression (blue) relative to uninfected controls. ns: not significant. *P < 0.05, **P < 0.01, ***P < 0.001, and ****P < 0.0001.

The immune response triggered by viral infection plays a critical role in establishing the host’s antiviral innate immunity (40). To compare route-dependent immune activation, 7-day-old BALB/c mice were infected with 1 LD_50_ of EV71-GZCII via either i.g. or i.p. Spleens and intestines were harvested from six mice per group at 1, 4, 8, and 12 dpi, and the mRNA expression levels of 28 cytokines associated with inflammation were measured using a relative quantitative method, with the housekeeping gene GAPDH serving as an internal control (Fig. 5; Fig. S3 and S4). The primers used are listed in Table S2 in the Supplement File. On the first day after oral infection, EV71 significantly induced the expression of cytokines such as CCL2, CCL3, CCL5, CXCL1, IFNγ, IL-1β, IL-4, IL-6, IL-10, IL-12-p40, iNOS, TLR9, and TRIF in the mouse spleen (Fig. 5A and C; Fig. S3). This indicates that EV71 infection triggers both TH1 and THαβ immune responses in the host. Among these cytokines, IL-12-p40 expression increased the most, by approximately 76-fold (Fig. 5A and C). Notably, on the first day after intraperitoneal infection, the expression of CCL3 in the spleen only increased by about 2-fold, whereas in orally infected conditions, CCL3 expression increased by approximately 12-fold (Fig. 5A and C). Surprisingly, no significant difference in intestinal cytokine expression was observed at 1 dpi (Fig. 5B and D; Fig. S4).

On the 4th day after oral infection, the expression levels of cytokines such as ADAR1, CCL2, CCL3, CCL5, CXCL1, IFNγ, IL-1β, IL-4, IL-6, IL-10, IL-12-p40, IRF7, MyD88, PKR, RIG-1, T-BET, and TLR9 were significantly upregulated in the mouse spleen (Fig. 5A and C; Fig. S3), indicating a more robust immune response. In contrast, on day 4 after intraperitoneal infection, there was no upregulation of ADAR1 and TLR9, and the expression levels of CXCL1 and IL-4 were downregulated (Fig. 5A and C; Fig. S3). Compared to day 1 post-oral infection, the upregulation of IRF7 at 4 dpi was the highest, increasing from 1.7-fold to 78.3-fold (Fig. 5A and C). Notably, significant upregulation of ADAR1 and PKR was not observed in intraperitoneally infected mice (Fig. 5A and C). At this stage, orally infected mice exhibited a significantly higher intestinal iNOS expression level compared to those infected via the intraperitoneal route (Fig. 5B and D).

On day 8 after oral infection, the expression levels of cytokines such as CCL2, CCL3, CCL5, IFNγ, IL-1β, IL-6, IL-12p40, IRF7, MyD88, NF-κB, T-BET, TGF-β1, TLR9, TNFα, and TRIF were significantly upregulated in the mouse spleen (Fig. 5A and C; Fig. S3). However, PKR and RIG-I expression, which were upregulated at 4 dpi, reverted to downregulation (Fig. 5A and C). This suggests that EV71 may encode specific proteins to interfere with host signaling pathways, inhibiting the expression or function of RIG-I and PKR, thereby extending its own replication cycle and promoting viral particle release. Consequently, the systemic viral RNA load in mice remained high at 8 dpi (Fig. 3). In contrast, intraperitoneally injected mice did not show upregulation of IL-6, MyD88, TNFα, and TRIF, although other cytokines, such as iNOS, were significantly upregulated (Fig. 5A and C; Fig. S3). A differential expression of TGF-β1 was similarly observed in the intestine (Fig. 5B and D). These differences in cytokine expression indicate that the route of infection can influence both the pathogenic mechanisms of the virus and the host immune response.

By day 12 post-oral infection, the expression levels of cytokines such as CCL2, CCL3, CCL5, IL-1β, IRF7, NFκB, T-BET, and TGF-β1 remained elevated in the spleen (Fig. 5A and C; Fig. S3). In contrast, only CCL5, iNOS, IRF7, T-BET, and TGF-β1 showed increased expression in intraperitoneally infected mice, with significantly lower levels of TGF-β1, NFκB, and TLR3 (Fig. 5A and C; Fig. S3). Additionally, intestinal TLR7 expression was elevated in the orally infected group (Fig. 5B and D). This indicates that, during the late stage of EV71 infection, orally infected mice remain in a state of chronic inflammation and immune suppression, characterized by disrupted immune metabolism, impaired tissue repair, and persistent viral replication, compared to intraperitoneally infected mice.

Overall, intraperitoneal infection resulted in excessive expression of inflammatory factors such as IL-12-p40, iNOS, and IRF7 in the spleen, with the most significant difference observed in iNOS mRNA expression (Fig. 5A and C), suggesting that i.p. infection may cause additional non-natural EV71 infection-related damage to the host. In contrast, oral infection exhibited higher levels of antiviral immune responses, involving ADAR1, NFκB, PKR, TGF-β1, and TLR3, as well as inflammatory responses mediated by CCL3 and TNFα, demonstrating a more comprehensive and sustained immune response.

### Evaluation of the therapeutic effect of rupintrivir on EV71-GZCII using the oral infection mouse model

**Fig 6:**
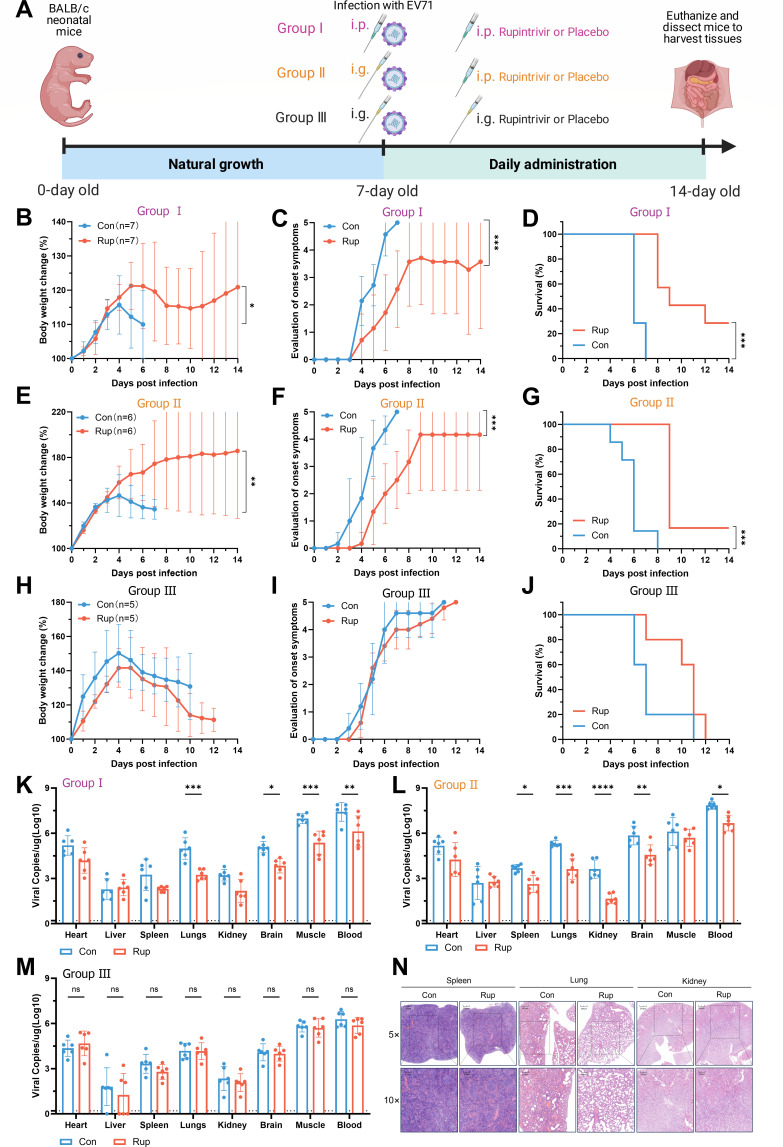
Evaluation of the therapeutic effect of rupintrivir on lethal EV71-GZCII challenge. (**A**) Schematic representation of the viral infection and therapeutic regimen in BALB/c mice. Changes in body weight (**B, E, and H**), clinical symptoms (**C, F, and I**), and survival curves (**D, G, and J**) in mice continuously administered Rup or Con after 10 LD_50_ EV71-GZCII challenge. (**K, L, and M**) Viral RNA loads in the heart, liver, spleen, lung, kidney, brain, muscle, and blood were detected in the Con or Rup groups at 7 dpi. The dashed line indicates the limit of detection. ns: not significant. *P < 0.05, **P < 0.01, ***P < 0.001, and ****P < 0.0001. (**N**) H&E staining of spleen, lung, and kidney. Scale bars: 200 μm (5× amplification) and 100 μm (10× amplification).

Rupintrivir, originally developed for the treatment of human rhinovirus (HRV) infections and currently undergoing Phase II clinical evaluation (41, 42), is a broad-spectrum antiviral compound that inhibits viral replication by targeting the 3C protease (43) and has demonstrated a favorable safety profile and pharmacokinetic properties. Notably, rupintrivir demonstrates strong binding affinity specifically for the EV71 3C protease (44, 45), positioning it as a promising therapeutic candidate for the treatment of EV71 infections. Using the EV71 oral infection model, we evaluated the *in vivo* therapeutic efficacy of rupintrivir against severe EV71 infection and further investigated the impact of infection route on treatment outcome. Specifically, 7-day-old BALB/c mice were randomly assigned to three experimental groups. In Group I, mice were challenged intraperitoneally with 10 LD₅₀ of EV71-GZCII and treated with rupintrivir (0.1 mg/kg) or vehicle via intraperitoneal injection. In Group II, mice were orally inoculated with 10 LD₅₀ of EV71-GZCII and subsequently treated with rupintrivir or vehicle via intraperitoneal administration. In Group III, mice were orally inoculated with 10 LD₅₀ of EV71-GZCII and treated with rupintrivir or vehicle by oral gavage. All mice were euthanized at 7 dpi, and tissues were collected for quantification of viral RNA loads (Fig. 6A).

In Group I, rupintrivir-treated (Rup) mice began to exhibit body weight loss at 6 dpi, followed by recovery at 10 dpi (Fig. 6B). Clinical symptoms peaked at 9 dpi with a mean clinical score of 3.71 (Fig. 6C), accompanied by a 28.57% increase in survival compared with control (Con) mice (Fig. 6D). Consistent with these clinical improvements, rupintrivir treatment resulted in a substantial reduction in viral RNA loads in the lungs, brain, muscle, and blood (Fig. 6K). In Group II, Rup mice maintained a gradual increase in body weight throughout the observation period (Fig. 6E).

Clinical symptoms reached maximal severity at 9 dpi, with a mean score of 4.17 (Fig. 6F). However, compared with Con mice, rupintrivir treatment conferred only a modest reduction in mortality (16.67%) (Fig. 6G). Nevertheless, viral RNA loads were significantly reduced in the spleen, lungs, kidneys, brain, and blood (Fig. 6L), and histopathological analysis revealed markedly attenuated pathological lesions in the spleen, lungs, and kidneys of the Rup group (Fig. 6N). In addition, the therapeutic efficacy of intraperitoneally administered rupintrivir against oral infection with 1 LD₅₀ EV71-GZCII was also evaluated (Fig. S5A). Compared with Con mice, Rup mice did not exhibit a significant improvement in body weight gain (Fig. S5B); however, a modest attenuation of clinical symptoms was observed (Fig. S5C), accompanied by a 20% increase in survival, although this difference did not reach statistical significance (Fig. S5D). Consistent with these findings, viral RNA loads were reduced only in the heart, lungs, muscle, and blood, with no significant decreases detected in other tissues (Fig. S5E). The contrasting therapeutic outcomes observed between infection routes are likely attributable to insufficient intestinal exposure to rupintrivir. In parallel, to determine whether orally administered rupintrivir could confer comparable therapeutic benefits in orally infected mice, EV71-GZCII–challenged animals were treated by oral gavage (Group III). Under these conditions, rupintrivir failed to exhibit any detectable therapeutic efficacy, as evidenced by unchanged body weight (Fig. 6H), clinical scores (Fig. 6I), survival rates (Fig. 6J), and tissue viral RNA loads (Fig. 6M). This lack of efficacy may result from degradation of rupintrivir within the gastrointestinal tract, leading to loss of biological activity, or from its inability to effectively traverse the intestinal barrier and reach sites of viral dissemination.

### Supplementation with intestinal probiotics inhibits replication and dissemination of EV71-GZCII in an oral infection mouse model

**Fig 7:**
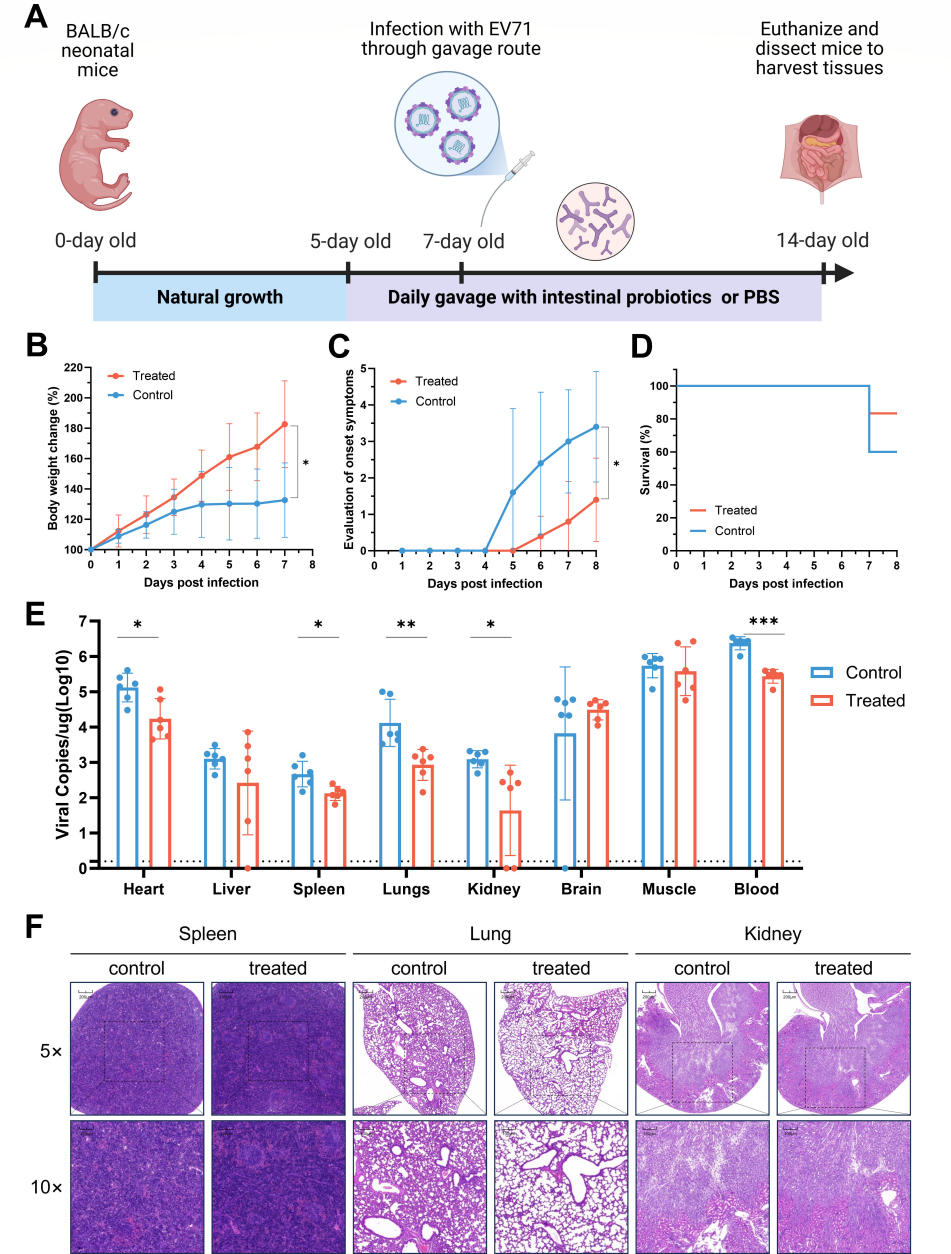
Evaluation of the therapeutic and alleviating effects of intestinal probiotics on low-dose EV71-GZCII infection using a mouse model of oral infection. (**A**) Schematic representation of the viral infection and therapeutic regimen in BALB/c neonatal mice. Changes in body weight (**B**), clinical symptoms (**C**), and survival curves (**D**) in mice following oral infection with 1 LD_50_ of EV71-GZCII (Control: *n* = 5; Treated: *n* = 5). (**E**) Viral RNA loads in the heart, liver, spleen, lung, kidney, brain, muscle, and blood were detected in the PBS (Control) or intestinal probiotics (Treated) groups at 7 dpi. The dashed line indicates the limit of detection. *P < 0.05, **P < 0.01 and ***P < 0.001. (**F**) H&E staining of spleen, lung, and kidney. Scale bars: 200 μm (5× amplification) and 100 μm (10× amplification).

Clinical studies have shown that the gut microbiota composition in children with HFMD undergoes significant alterations, with a marked reduction in beneficial bacteria such as Bifidobacterium and Bacteroides (46). Based on the established pathophysiology of EV71 infection and the well-characterized biological functions of intestinal probiotics, intestinal probiotic blockade therapy is anticipated to hold significant potential for mitigating EV71 infection. Remarkably, only the oral infection model can accurately evaluate the efficacy of such intestinal probiotic interventions, whereas intraperitoneal infection models bypass gut-specific pathways. Accordingly, we assessed the anti-EV71 activity of a clinically used prescription probiotic formulation, Siliankang—a combination of *Bifidobacterium infantis*, *Lactobacillus acidophilus*, *Enterococcus faecalis*, and *Bacillus cereus*—commonly employed for correcting dysbiosis. From 5 days of age, BALB/c neonatal mice were daily gavaged with either an intestinal probiotic suspension (Treated group) or PBS (Control group) until the end of the experiment. At 7 days of age, both groups were orally challenged with 1 LD_50_ of EV71-GZCII (Fig. 7A). At 7 dpi (when mice were 14 days old), six mice from each group were euthanized, and tissues were harvested for viral load analysis.

Compared to the Control group, the Treated group exhibited faster overall body weight gain (Fig. 7B), milder clinical symptoms (Fig. 7C), and improved survival rates (Fig. 7D). Importantly, the Treated group exhibited markedly reduced viral loads in several organs, including the heart, spleen, lungs, kidneys, and blood (Fig. 7E). Similarly, markedly attenuated pathological changes in the spleen, lungs, and kidneys were also observed in the treated group (Fig. 7F). These results demonstrate that probiotic therapy confers protective effects against EV71 oral infection, and further indicate that the oral infection model serves as a robust tool for assessing the efficacy of therapeutics targeting intestinal blockade.

## DISCUSSION

EV71 primarily spreads via the fecal-oral route, predominantly affecting infants and young children under 5 years of age. It can cause HFMD with mild symptoms such as fever, oral ulcers, and vesicular rashes (5). However, severe neurological complications like encephalitis, meningitis, and acute flaccid paralysis can also occur, leading to high morbidity and mortality. EV71 accounts for about 80% of severe HFMD cases and 90% of related fatalities in China (19), making it a critical health concern. Understanding the pathogenicity and mechanisms of EV71 is crucial, but the lack of effective animal models simulating natural infection has hindered research progress. Thus, the establishment of an oral EV71 infection model in immunocompetent mice is essential for investigating viral pathogenesis and for the preclinical development of antiviral therapeutics.

The EV71-GZCII strain was derived from an EV71-infected child hospitalized at Guangzhou Children’s Hospital, China (3). Previous studies have shown that intraperitoneal inoculation of EV71-GZCII in mice leads to severe neurological symptoms and high mortality, demonstrating robust murine pathogenicity that positions this strain as a candidate model for oral infection studies (3, 33). Our results demonstrated that oral inoculation of 7-day-old immunocompetent BALB/c mice with 5 × 10^7^ TCID₅₀ of EV71-GZCII strain resulted in robust infection, characterized by severe neurological manifestations, including ataxia and limb paralysis, culminating in 100% mortality. This represents the first report of a clinically derived EV71 strain causing lethal oral infection in immunocompetent mice, overcoming limitations of prior models relying on intraperitoneal inoculation of laboratory-adapted variants (27, 29, 34, 37, 47), which poorly recapitulate natural enteric transmission.

The intraperitoneal infection route evades gastrointestinal barriers, enabling direct systemic viral dissemination, whereas oral infection must first overcome the gastrointestinal mucosal and immune defenses. Although both routes induced substantial viremia, these observations indicate that the route of infection profoundly influences EV71 transmission dynamics and mechanisms of CNS entry. Notably, in our oral infection model, EV71 was detected in the brain as early as 1 dpi, preceding its detection in skeletal muscle. This early CNS involvement has not been reported in previous intraperitoneal or oral infection studies employing mouse-adapted EV71 strains or transgenic mouse models. However, a similar phenomenon has been observed following direct injection of EV71 into unilateral jaw or facial muscles, where viral antigen or RNA was detected in the CNS as early as 1 dpi (48). Based on our findings and previous reports, we speculate that EV71 infection of the intestinal tract may transiently increase blood-brain barrier (BBB) permeability (49), allowing viral entry into the brain at early time points. Alternatively, orally acquired virus may gain access to the CNS through infection and activation of intestinal endothelial cells (50). Such gut-dependent routes of neuroinvasion are bypassed in intraperitoneal infection models. However, the presence of virus in the brain at early time points did not correlate with disease severity or clinical outcome, suggesting that hematogenous dissemination alone may be insufficient to account for EV71 neuroinvasion (49). During the middle to late stages of infection, subsequent viral dissemination to skeletal muscle and robust replication at this site may support sustained viral persistence, serving as a continuous source of virus for hematogenous spread and CNS invasion (25, 51, 52). Our data therefore favor a predominantly blood-borne route of neuroinvasion, while not excluding alternative mechanisms such as retrograde axonal transport, which warrant further systematic investigation. In contrast, despite comparable levels of viremia early after intraperitoneal infection, viral loads in the brain were lower than those observed following oral challenge, potentially due to limited modulation of BBB permeability when the intestinal tract is bypassed. After 4 dpi, EV71 successfully invaded and replicated in the intestine, coinciding with a further increase in BBB permeability and a marked rise in brain viral burden. Skeletal muscle may also act as an initial replication site, contributing to CNS invasion during the mid-to-late stages of infection. The differences in viral replication and dissemination between the two routes underscore the necessity of establishing a reliable mouse model for oral EV71 infection. Although the same LD_50_ of EV71-GZCII was used for infection, oral infection required a markedly higher TCID₅₀ inoculum than intraperitoneal infection (i.p., 2.7 × 10^3^ TCID_50_; i.g., 1.8 × 10^6^ TCID_50_), consistent with observations reported in previous studies (49). This implies that, despite the presence of three mutations that enhance virulence, EV71 still faces challenges in naturally infecting mice, likely due to the immune barrier function of the gut and the inefficiency of viral transmission from the gut to the CNS (29). In contrast, during intraperitoneal infection, EV71 circumvents the intestinal immune barrier, which may facilitate broader viral dissemination. However, it is important to note the limitations of conclusions drawn from this infection model.

At present, the clinical management of EV71 infection remains largely supportive, as no antiviral agents have received regulatory approval. Among investigational therapies, rupintrivir has garnered considerable attention as a promising candidate with potential therapeutic efficacy against EV71. In this study, we demonstrate that the therapeutic efficacy of rupintrivir is markedly influenced by the route of viral infection. The diminished efficacy of rupintrivir in oral infection models relative to intraperitoneal models primarily reflects fundamental differences in viral entry pathways, the drug’s pharmacokinetic profile, and host microenvironmental responses. In intraperitoneal infection, EV71 circumvents the intestinal barrier, directly accessing the peritoneal circulation and systemic bloodstream to rapidly establish infection in target organs. This route coincides with rupintrivir’s systemic distribution, allowing the drug to reach therapeutic concentrations before extensive viral replication occurs and to act synergistically with the rapidly mobilized systemic immune response, thereby enhancing antiviral efficacy. In contrast, oral EV71 infection necessitates viral persistence within the intestinal tract to overcome physiological barriers, including the mucus layer, intestinal epithelium, and resident gut microbiota, resulting in a prolonged phase of local viral retention. Rupintrivir—whether administered orally, subject to gastric degradation, first-pass metabolism, and microbial biotransformation, or delivered parenterally, with limited penetration across the blood-intestinal barrier—fails to reach sufficient concentrations at the intestinal mucosa, the primary site of viral replication. Moreover, localized intestinal mucosal immunity, such as secretory IgA, delays systemic immune activation, abrogating potential synergistic effects between the host immune response and the antiviral drug. The spatially dispersed nature of viral replication further reduces the probability of effective drug-virus interactions. Collectively, these findings underscore the critical value of oral infection models that faithfully recapitulate the natural route and physiological context of EV71 infection.

Intestinal probiotics have considerable potential to inhibit EV71 infection by strengthening gut barrier integrity, modulating both local and systemic immune responses, and competitively blocking viral attachment. Importantly, the oral infection model is indispensable for evaluating probiotic efficacy, as it faithfully recapitulates the natural route of viral entry and initial replication within the gastrointestinal tract—an advantage not afforded by intraperitoneal models. This physiologically relevant system enables rigorous assessment of key probiotic-virus interactions, including competition for epithelial binding sites, reinforcement of the intestinal mucosal barrier, and induction of secretory IgA responses, all of which are avoided in parenteral infection models. Accordingly, only oral challenge studies can reliably predict the protective potential of probiotics in clinically meaningful settings. In this study, utilizing the EV71 clinical isolate oral infection model newly established in BALB/c mice, we evaluated the antiviral efficacy of an intestinal probiotic formulation for the first time. Our findings demonstrate that intestinal probiotics attenuate low-dose EV71 infection by suppressing viral replication and systemic dissemination, suggesting their potential in preventing EV71 infection and managing mild or early-stage disease, potentially serving as adjuncts to antiviral therapies. However, strategies to selectively target and enhance these antiviral effects require further investigation to fully realize their clinical potential. In addition to providing insights into therapeutic development, this study highlights the utility of the EV71 oral infection model for preclinical drug evaluation. Although the relationship between viral genomic mutations and pathogenicity was not extensively explored here, the established model remains a valuable platform for future studies on EV71 pathogenesis.

In summary, we have successfully developed an oral infection model in immunocompetent mice using the clinical EV71-GZCII isolate, revealing distinct viral dissemination patterns and host immune responses between intraperitoneal and oral infection routes. This clinical isolate-based oral model overcomes critical limitations of conventional systems, such as high economic costs, low infection efficacy, and poor replication of natural transmission routes. An indispensable advantage of this model is its unique capacity to evaluate intestinal probiotic blockade therapy, which cannot be accurately assessed using intraperitoneal infection models that circumvent the gastrointestinal tract. Utilizing this oral infection system, we demonstrated that probiotic formulations significantly reduce viral loads and mitigate disease progression, highlighting their translational potential for clinical intervention. Consequently, the established oral EV71 infection model not only provides a rigorous platform for elucidating viral pathogenesis and host-virus interactions but also serves as an essential tool for validating novel antiviral strategies, particularly those targeting the intestinal microenvironment, thereby facilitating the development of effective therapeutics against EV71 infection.

## MATERIALS AND METHODS

### Cells and viruses

RD (human rhabdomyosarcoma) and Vero (African green monkey kidney) cells were cultured in Dulbecco’s modified Eagle’s medium (DMEM, Gibco) supplemented with 10% fetal bovine serum (FBS, Gibco) at 37°C in a 5% CO_2_ incubator. The highly pathogenic EV71 strain EV71-GZCII, isolated from clinical specimens (3), was utilized in this study. The infectious cDNA clone pEV71-GZCII was generated, and the recombinant virus was rescued as previously described (33). Complete nucleotide sequence data for EV71-GZCII strain are available in GenBank under accession number MN171486.1. Vero cell monolayers were infected with viral inoculum at a multiplicity of infection (MOI) of 0.01, followed by a 2-hour adsorption period at 37°C. After the removal of the viral inoculum, cells were maintained in DMEM supplemented with 2% FBS at 37°C in a 5% CO_2_ incubator. Viral propagation was monitored daily until approximately 80% cytopathic effect was observed under light microscopy. Culture supernatants were collected, subjected to three freeze-thaw cycles at −80°C, and clarified by centrifugation (8,000 × *g*, 4°C, 10 minutes). Virus titers were determined in Vero or RD cells using the Reed and Muench method (53).

### Animal experiment

NOD/SCID and A129 mice were bred under specific-pathogen-free conditions at the institutional animal facility, while pregnant BALB/c mice were commercially sourced. Throughout the experiment, the suckling mice remained with their dam and were nursed naturally. Mice were randomly assigned to experimental groups and were humanely euthanized upon reaching predefined humane endpoints, including complete limb paralysis or the development of severe and irreversible clinical signs.

Mice were inoculated intraperitoneally or via oral gavage with EV71 viral suspensions or PBS upon reaching the designated postnatal age. Clinical outcomes were evaluated daily for 14 dpi. The severity of clinical symptoms was scored on a scale of 0–5, where 0 = normal movement and healthy, 1 = ruffled fur or hunchback, decreased activity, hypersomnolence, 2 = limb weakness, 3 = limb paralysis, 4 = near death, and 5 = death.

At 1, 4, 8, and 12 dpi, mice from each experimental group were euthanized, and major organs, including heart, liver, spleen, lung, kidney, intestine, brain, muscle tissue, and blood, were harvested under aseptic conditions. Tissue homogenates were analyzed for viral RNA load by quantitative RT-PCR. Parallel samples were formalin-fixed for histopathological evaluation of virus-induced lesions.

For therapeutic efficacy assessment, 7-day-old BALB/c mice were challenged with 10 LD₅₀ of EV71-GZCII via either i.p. or i.g. route. In the treatment group (Rup), rupintrivir (MedChemExpress) was administered at a dose of 0.1 mg/kg, formulated in 25 μL of vehicle containing 0.2% DMSO and 99.8% sterile saline, once daily for 10 consecutive days. Control mice (Con) received an equivalent volume of vehicle alone following the same dosing schedule. Mice were monitored daily for changes in body weight, clinical manifestations, and survival status. At 7 dpi, the mice were euthanized, and major organs were collected.

In the experiment on the prevention and treatment of intestinal probiotics, starting on postpartum day 5, the Control group received daily intragastric administration of PBS, while the Treated group was administered a combined probiotic formulation (Siliankang), containing *Bifidobacterium infantis*, *Lactobacillus acidophilus*, *Enterococcus faecalis*, and *Bacillus cereus*, suspended in PBS at a dose of 6.2 × 10³ CFU per day. On day 7 postpartum, mice were intragastrically infected with 1 LD_50_ of EV71-GZCII, and their symptoms were monitored daily. At 7 dpi, mice were sacrificed, and major organs were harvested.

### Extraction and detection of viral RNA

Total RNA was isolated from 200 μL of blood or tissue homogenates using the AFTSpin Viral DNA/RNA Extraction Kit (ABclonal); RNA integrity and concentration were assessed spectrophotometrically using a NanoDrop 2000 (Thermo Fisher Scientific). Quantitative reverse transcription PCR (qRT-PCR) was performed using the HiScript II One Step qRT-PCR Probe Kit (Vazyme), with primers EV71-RT-F and EV71-RT-R targeting the VP1 capsid protein-coding region (primer sequences in Table S2). Viral RNA copy numbers per µg of total RNA were determined using a standard curve generated from serial dilutions of pEV71-GZCII containing the full-length VP1 gene.

To quantify inflammatory factor mRNA levels in the spleen and intestine of BALB/c mice after various treatments, total RNA was extracted using the AFTSpin Viral DNA/RNA Extraction Kit (ABclonal). First-strand cDNA synthesis was carried out with the HiScript III 1st Strand cDNA Synthesis Kit (Vazyme), followed by quantitative PCR (qPCR) using ChamQ Universal SYBR qPCR Master Mix (Vazyme). Target mRNA levels (primer sequences in Table S2) were normalized to the housekeeping gene GAPDH and calculated using the 2^−∆∆Ct^ method. Data are expressed as fold-changes relative to uninfected controls.

### Histopathological analysis

Tissues obtained from infected and uninfected BALB/c mice were fixed in 4% paraformaldehyde for 48 hours, followed by dehydration through a graded ethanol series and paraffin embedding. Tissue sections were stained with H&E, and images were observed under a microscope and analyzed.

### Statistical analysis

All experiments were conducted at least three times. Data were analyzed using GraphPad Prism 9 (CA, USA) and expressed as mean ± standard deviation (SD). Two-way ANOVA was used for comparing multiple groups, while Student’s t-tests were used to compare two groups. A *P*-value < 0.05 was considered statistically significant (*P < 0.05, **P < 0.01, ***P < 0.001, and ****P < 0.0001).

## Data Availability

All data needed to evaluate the results are included in the article and its supplemental material.

## SUPPLEMENTAL MATERIAL

Supplemental material jvi.02068-25-s0001.docx Tables S1 and S2; Fig. S1 to S5.

Graphical abstract jvi.02068-25-s0002.tif Visual depiction of the study.

ASM does not own the copyrights to Supplemental Material that may be linked to, or accessed through, an article. The authors have granted ASM a non-exclusive, world-wide license to publish the Supplemental Material files. Please contact the corresponding author directly for reuse.
