# Supplementary material for: Establishment of an oral enterovirus 71 (EV71) infection model in immunocompetent mice for antiviral therapy evaluation
Source: J Virol. 2026 May 21;100(6):e02068-25. doi: 10.1128/jvi.02068-25 (PMC13288771; doi:10.1128/jvi.02068-25)
Supplement: Supplemental material — Tables S1 and S2; Fig. S1 to S5. [file jvi.02068-25-s0001.docx]

**Establishment of an oral enterovirus 71 (EV71) infection model in immunocompetent mice for antiviral therapy evaluation**

Yisha Ma^1,2^, Dan Luo^3^, Xianliang Ke^1,2^, Xiaohui Song^4^, Mengchan Hao^1,2^, Jianjun Chen*^1,2^, and Yuan Zhang*^1,2^

1. State Key Laboratory of Virology and Biosafety, Wuhan Institute of Virology, Chinese Academy of Sciences, Wuhan 430071, People's Republic of China.

2. University of Chinese Academy of Sciences, Beijing 100049, People's Republic of China.

3. Department of Gastroenterology, Wuhan Children’s Hospital, Tongji Medical College, Huazhong University of Science and Technology, Wuhan, Hubei Province, People’s Republic of China.

4. Department of Obstetrics, Wuhan Children's Hospital, Wuhan Maternal and Child Healthcare Hospital, Tongji Medical College, Huazhong University of Science & Technology, Wuhan, 430015, China.

*Corresponding author: Jianjun Chen (chenjj@wh.iov.cn) and Yuan Zhang (zhangyuan@wh.iov.cn)

Table S1

| Route | Dose (TCID_50_) | Mortality |
| --- | --- | --- |
| intraperitoneal | 10^2^ | 0% (0/5) |
|  | 10^3^ | 25% (2/8) |
|  | 10^4^ | 83.33% (5/6) |
|  | 10^5^ | 100% (5/5) |
| intragastric | 10^5^ | 0% (0/5) |
|  | 10^6^ | 33.33 (2/6) |
|  | 10^7^ | 100% (6/6) |

Table S1. Mortality of mice infected with EV71-GZCII via different routes and at different inoculation doses.

Table S2

Summary of Primer Sequences

| Primer name | Primer sequence |
| --- | --- |
| EV71-RT-F | AAATGGCAGCGCCACTGAAG |
| EV71-RT-R | GGGCGCGTTGGTTTATCCAC |
| GAPDH-F | CAACGGCACAGTCAAGGCCG |
| GAPDH-R | CTCCATGGTGGTGAAGACAC |
| ADAR1-F | AATCTCTGCCCTGCTCCTTT |
| ADAR1-R | CTGGCTGGTTGATCTGATGC |
| CCL2-F | CAGCTCTCTCTTCCTCCACC |
| CCL2-R | TGGGATCATCTTGCTGGTGA |
| CCL3-F | CCAGCCAGGTGTCATTTTCC |
| CCL3-R | AGGCATTCAGTTCCAGGTCA |
| CCL5-F | TGCCAACCCAGAGAAGAAGT |
| CCL5-R | AGATGCCCATTTTCCCAGGA |
| CXCL1-F | TTGTATGGTCAACACGCACG |
| CXCL1-R | ACGAGACCAGGAGAAACAGG |
| GATA3-F | TCTCCAAGTGTGCGAAGAGT |
| GATA3-R | TCCGGATTCAGTGGTTGGAA |
| IL-1β-F | ACTCATTGTGGCTGTGGAGA |
| IL-1β-R | TTGTTCATCTCGGAGCCTGT |
| IL4-F | GAGAGTGAGCTCGTCTGTAG |
| IL4-R | ACTAGAGTTCTTCTTCAAGC |
| IL6-F | CATCCAGTTGCCTTCTTG |
| IL6-R | ATTAAGCCTCCGACTTGT |
| IL10-F | GCATGGCCCAGAAATCAAGG |
| IL10-R | AATCGATGACAGCGCCTCAG |
| IL12-p40-F | CAGAAGCTAACCATCTCCTGGTTTG |
| IL12-p40-R | TCCGGAGTAATTTGGTGCTTCACAC |
| IFNγ-F | CATGGCTGTTTCTGGCTGTT |
| IFNγ-R | TCCTTTTGCCAGTTCCTCCA |
| iNOS-F | CCCCGCTACTACTCCATCAG |
| iNOS-R | CCACTGACACTTCGCACAAA |
| IRF3-F | CTGAAAACCGTGGACTTGCA |
| IRF3-R | AGTCCATGTCCTCCACCAAG |
| IRF7-F | TGCTGTTTGGAGACTGGCTA |
| IRF7-R | CGAAATGCTTCCAGGGTACG |
| Myd88-F | ACATCTTTGACTCCCCTGGG |
| Myd88-R | CATGTGTGTACTGAGGTGCG |
| NFκB-F | CACCGGATTGAAGAGAAGCG |
| NFκB-R | AGTTGAGTTTCGGGTAGGCA |
| PKR-F | AGATTTCAGAGCCTGCACCT |
| PKR-R | TGGGGTATCACTGGCCATTT |
| RIG-1-F | CACTTCGTTCATCTCTGGCG |
| RIG-1-R | AGCCTGAATGTACTGCACCT |
| T-BET-F | GTGTCTGGGAAGCTGAGAGT |
| T-BET-R | GGTGAAGGACAGGAATGGGA |
| TGF-β1-F | TCGCTTTGTACAACAGCACC |
| TGF-β1-R | ACTGCTTCCCGAATGTCTGA |
| TLR2-F | CTGAGAATGATGTGGGCGTG |
| TLR2-R | TTAAAGGGCGGGTCAGAGTT |
| TLR3-F | TTGCGTTGCGAAGTGAAGAA |
| TLR3-R | TGTTCAAGAGGAGGGCGAAT |
| TLR4-F | GGAGACTGGGGAGCCATATC |
| TLR4-R | CAAGGACAACAGCACACCAA |
| TLR7-F | GCACTCTTCGCAGCAACTAA |
| TLR7-R | TCTTCCGTGTCCACATCGAA |
| TLR9-F | CAAGTACACGCTCAGATGGC |
| TLR9-R | CCCACTGATGCGATTGTCTG |
| TNFα-F | TGAGGTCAATCTGCCCAAGT |
| TNFα-R | GGGGTCAGAGTAAAGGGGTC |
| TRIF-F | GCCGGCTCCTGTTTTGTAAA |
| TRIF-R | TAGGGCTGCAGAAGTGTACC |

Figure S1


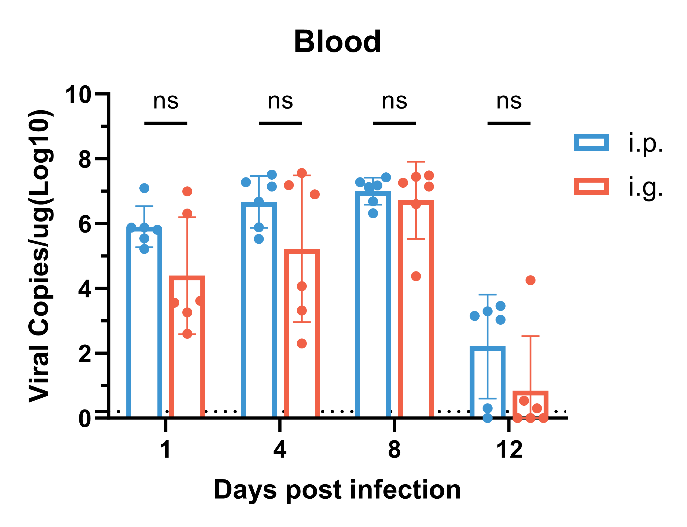


**Figure S1. Viral RNA loads in the blood of EV71-GZCII-infected mice via intraperitoneal or oral routes.** 7-day-old BALB/c mice were inoculated with 1 LD_50_ of EV71-GZCII through either intraperitoneal (i.p.) or oral (i.g.) routes. Blood samples were collected at 1, 4, 8, and 12 dpi for viral RNA quantification by RT-qPCR. The dashed line represents the limit of detection. ns: not significant.

Figure S2

| 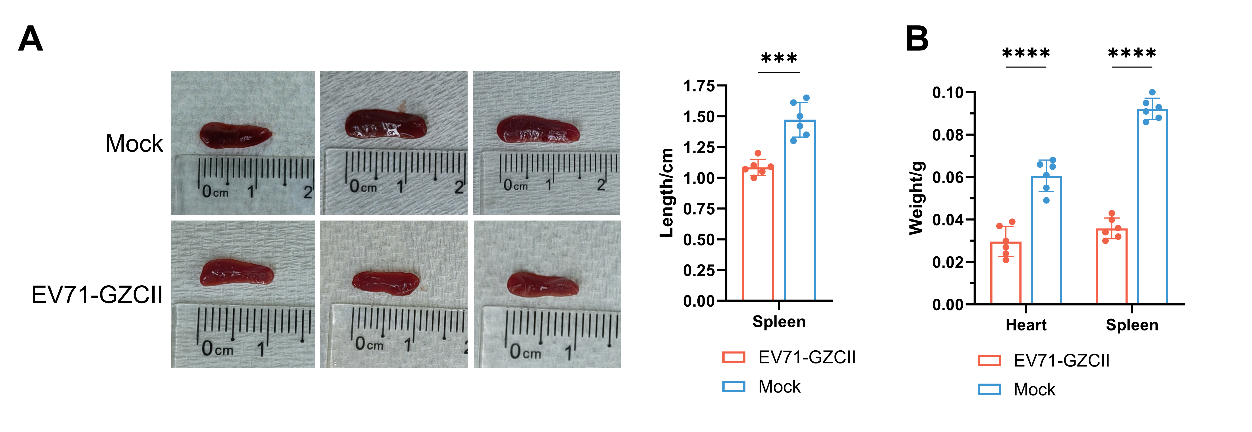 |
| --- |
| **Figure S2. Systemic histopathology induced by EV71-GZCII infection in BALB/c neonates.** Mock: uninfected mice, EV71-GZCII: orally infected mice. (A) Splenic atrophy: marked reduction in spleen longitudinal length in symptomatic mice following oral EV71 infection (***p < 0.001). (B) Organ hypoplasia: significant decreases in heart mass and spleen mass (****p < 0.0001). |

Figure S3

| 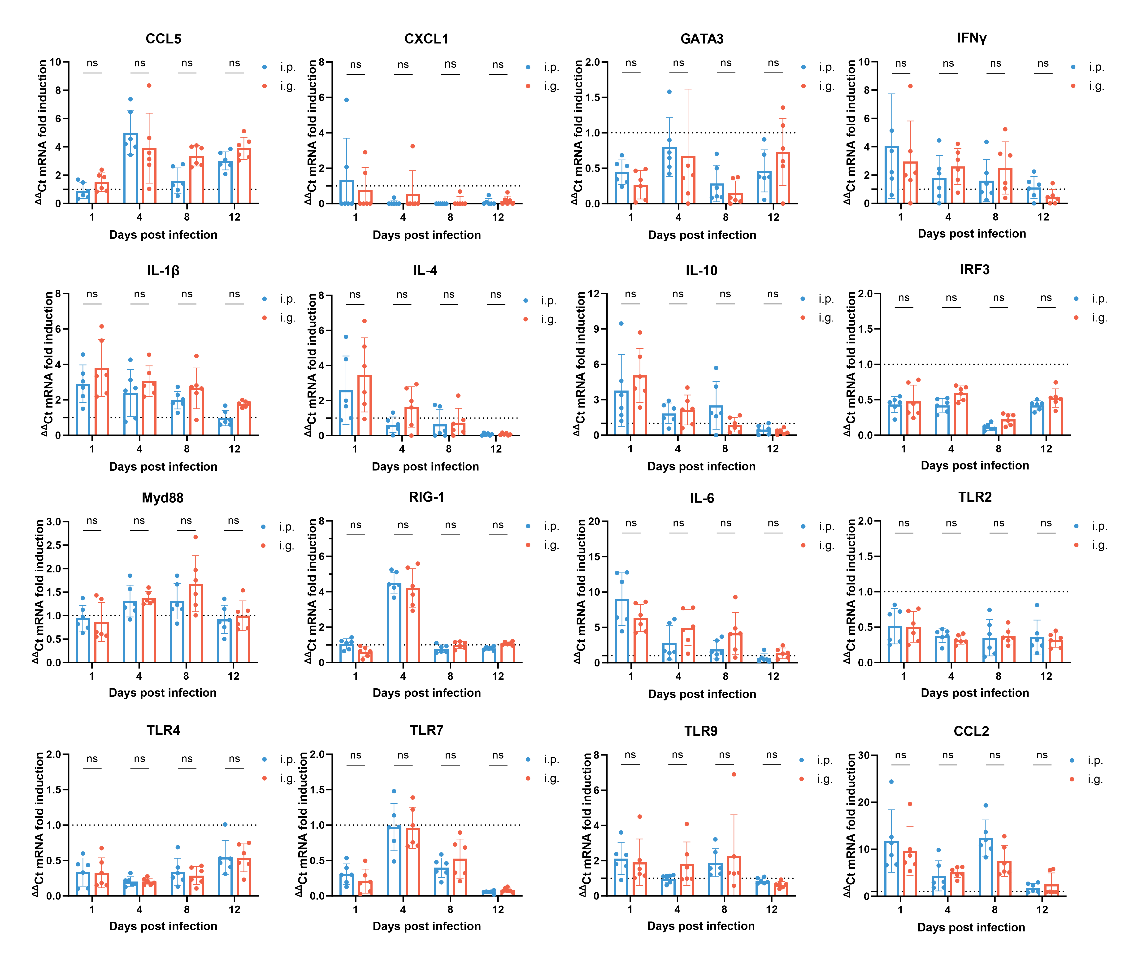 |
| --- |
| **Figure S3. Splenic cytokine dysregulation following EV71-GZCII infection.** Temporal cytokine mRNA profiles in the spleen of inoculated BALB/c via intragastric (i.g.) or intraperitoneal (i.p.) routes (1 LD_50_). Relative cytokine expression was normalized to GAPDH using the 2^⁻∆∆Ct^ method. The dashed line represents baseline levels in mock-infected controls. ns: not significant. |

Figure S4


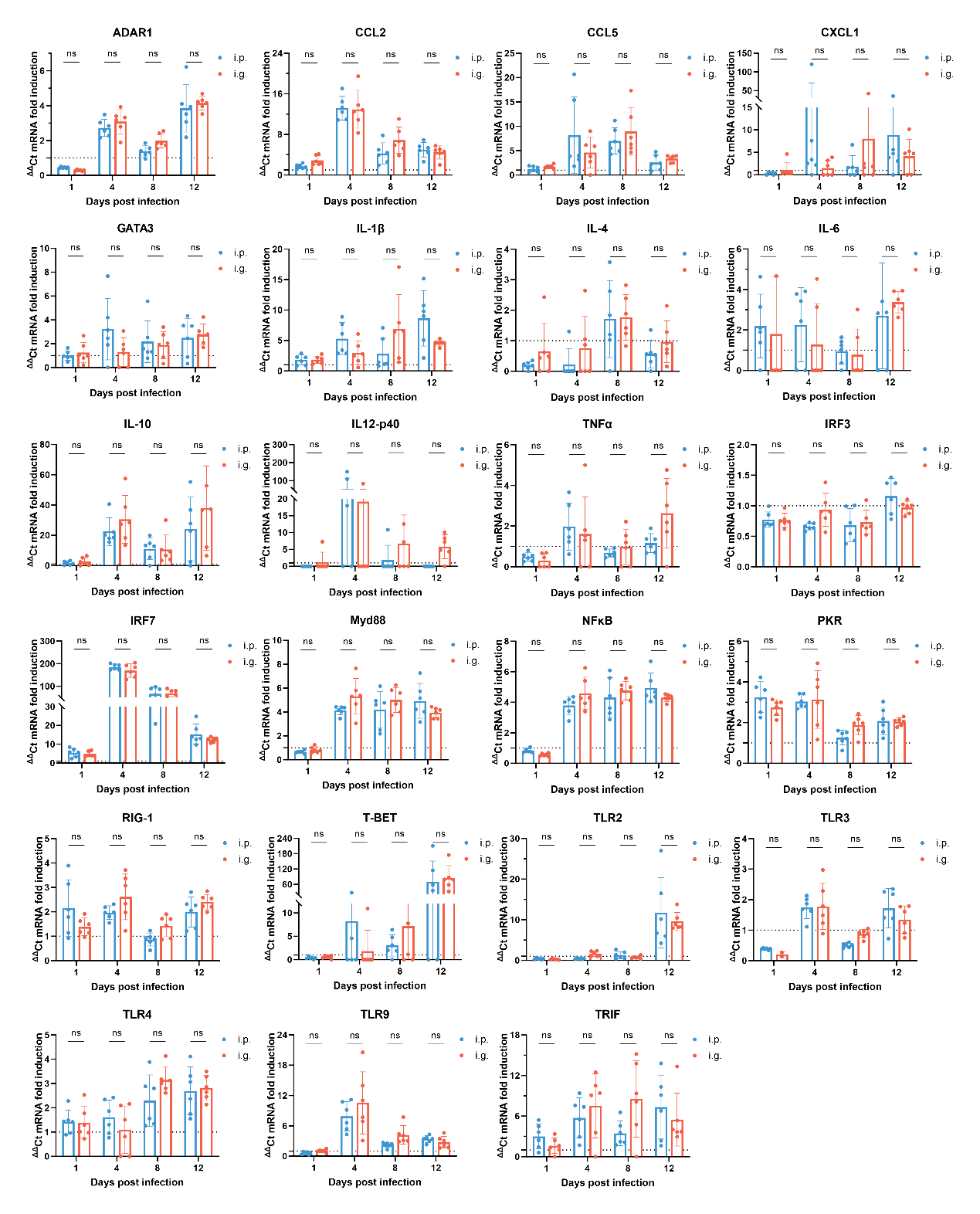


**Figure S4. Intestinal cytokine dysregulation following EV71-GZCII infection.** Temporal cytokine mRNA profiles in the intestine of inoculated BALB/c via intragastric (i.g.) or intraperitoneal (i.p.) routes (1 LD_50_). Relative cytokine expression was normalized to GAPDH using the 2^⁻∆∆Ct^ method. The dashed line represents baseline levels in mock-infected controls. ns: not significant.

Figure S5


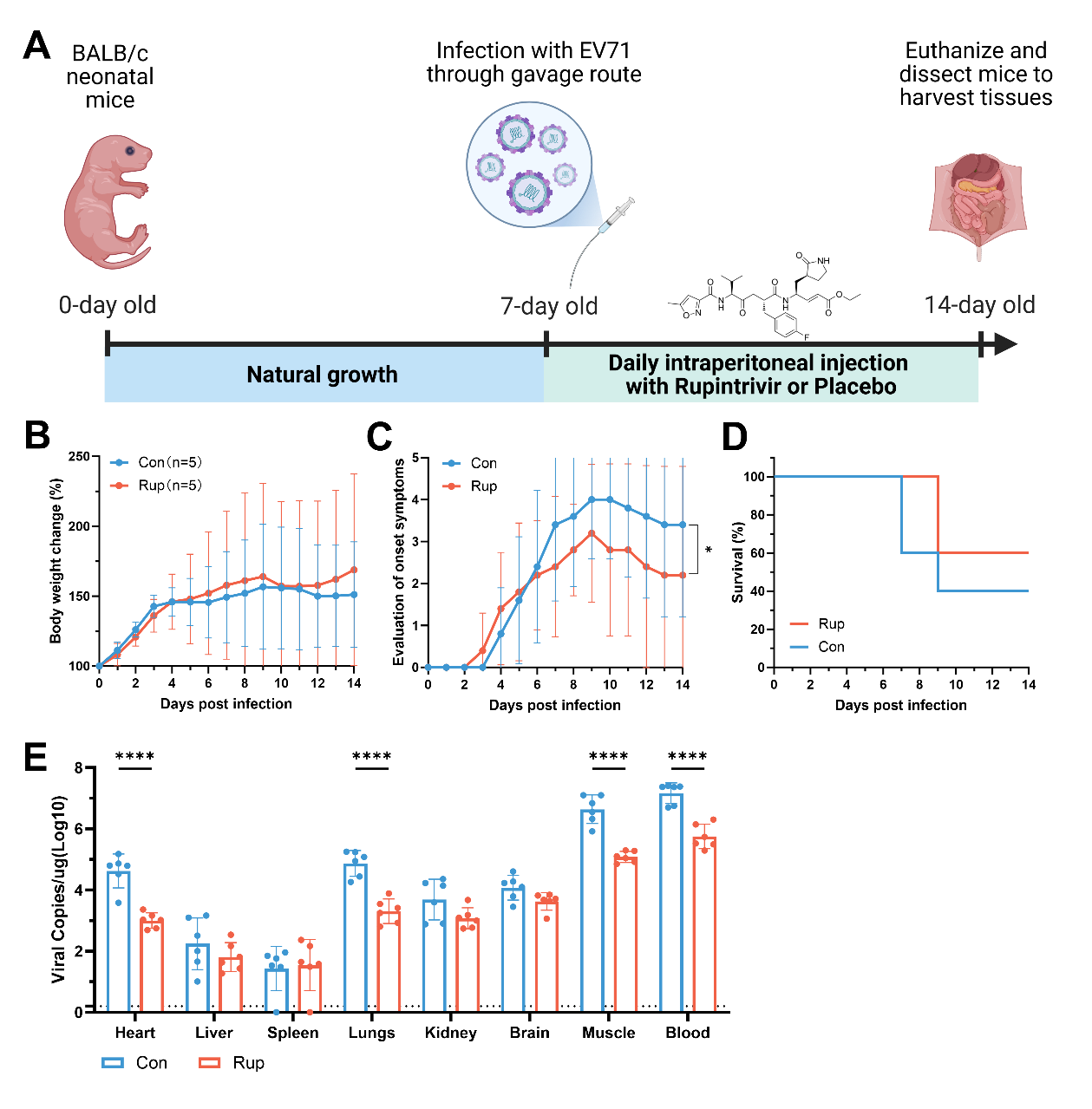


**Figure S5. Therapeutic efficacy of rupintrivir against EV71 oral infection in mice.** (A) 7-day-old BALB/c mice were infected intragastrically with 1 LD₅₀ EV71-GZCII and treated with rupintrivir via intraperitoneal administration (Con, n=5; Rup, n=5). Changes in body weight (B), clinical scores (C), survival curves (D), and viral RNA loads in indicated tissues (E) are shown. The dashed line indicates the limit of detection. *p < 0.05, ****p < 0.0001.
