# Supplementary figures and images for: Establishment of an oral enterovirus 71 (EV71) infection model in immunocompetent mice for antiviral therapy evaluation
Source: J Virol. 2026 May 21;100(6):e02068-25. doi: 10.1128/jvi.02068-25 (PMC13288771; doi:10.1128/jvi.02068-25)

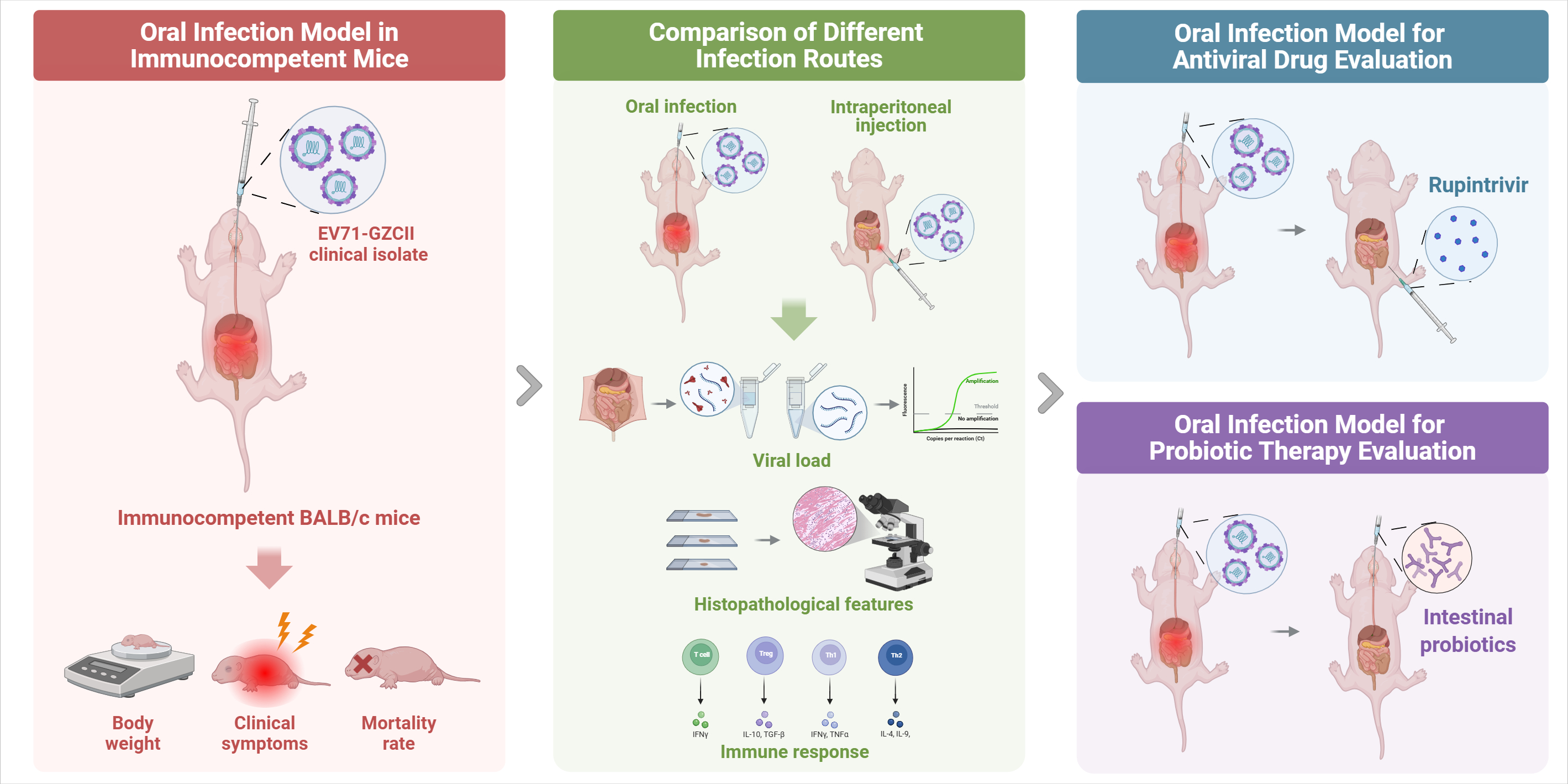

Supplement: Graphical abstract — Visual depiction of the study. [file jvi.02068-25-s0002.tif]
